# Mayfield-Tyzzer Hospital for Men at Laichon Fu, China

**Published:** 1911-04

**Authors:** J. M. Gaston

**Affiliations:** Physician in Charge


### Journal-Record of Medicine

Successor to Atlanta Medical and Surgical Journal, Established 1855, and Southern Medical Record, Established 1870.

OWNED BY THE ATLANTA MEDICAL JOURNAL CO.

Published Monthly

*Official Organ Fulton County Medical Society, State Examining Board, Presbyterian Hospital, Atlanta, Birmingham and Atlantic Railroad Surgeons' Association, Chattahoochee Valley Medical and Surgical Association, Etc.*

EDGAR G. BALLENGER, M. D., Editor.
BERNARD WOLFF, M. D., Supervising Editor.
A. W. STIRLING, M. D., C. M., D. P. H., J. S. HURT, B. Ph., M. D.
GEO. M. NILES, M. D., W. J. LOVE, M. D., (Ala.); Associate Editors.
E. W. ALLEN, Business Manager.

COLLABORATORS

Dr. W. F. WESTMORLAND, General Surgery.
F. W. McRAE, M. D., Abdominal Surgery.
H. F. HARRIS, M. D., Pathology and Bacteriology.
E. B. BLOCK, M. D., Diseases of the Nervous System.
MICHAEL HOKE, M. D., Orthopedic Surgery.
CYRUS W. STRICKLER, M. D., Legal Medicine and Medical Legislation.
E. C. DAVIS, A. B., M. D., Obstetrics.
E. G. JONES, A. B., M. D., Gynecology.
R. T. DORSEY, Jr., B. S. M. D., Medicine.
L. M. GAINES, A. B., M. D., Internal Medicine.
J. N. LeCONTE, M. D., Disease of the Stomach and Intestines.
L. B. CLARKE, M. D., Pediatrics.
EDGAR PAULIN, M. D., Opsonic Medicine.
THEODORE TOEPEL, M. D., Mechano Therapy.
R. R. DALY, M. D., Medical Society.
A. W. STIRLING, M. D., etc., Diseases of the Eye, Ear, Nose and Throat.
BERNARD WOLFF, M. D., Diseases of the Skin.
E. G. BALLENGER, M. D., Diseases of the Genito-Urinary Organs.

VOL. LVII. APRIL, 1911 NO. 1.

### MAYFIELD-TYZZER HOSPITAL FOR MEN AT LAI-CHON FU, CHINA.

BY J. M. GASTON, A. M., M. D., *Physician in Charge.*

In looking over a year's work it occurs to me that some cases treated would be of interest to friends in Georgia.

Human nature in China has many points of similarity to human nature in America; but some interesting differences may be noted.

The Chinese, when treated, as in patients of the Hospital, have usually been docile and grateful.

Their ignorance in regard to the potency of medicines has caused some amusing, not to say alarming circumstances. For instance, a man who wanted to get well quickly, took a whole bottle of salicylate of soda in solution, at one dose, when it was to have been given to him in small doses. He suffered no more than a temporary deafness therefrom.

The nervous system of the Chinese is less highly organized than that of the Anglo-Saxon. They are much less excitable and irritable. The patients who have taken a general anaesthetic have submitted well both to chloroform and ether. Except in cases of opium smokers hypodermic injections of morphine were given a half-hour before the administration of the anesthetic. In the opium smokers I have used strychnine sulphate as a stimulant. As a local anaesthetic, I have used cocaine with no untoward result. Even when no anaesthetic is given the Chinese bear pain with no complaint. This is especially true in the extraction of teeth, not a few of which have needed more than one trial. To the Chinese of this section, surgery is practically new; but their faith in it is great, and they do not hesitate, short of the loss of a member.

Their sense of humor is good, and helps them over many a situation which would otherwise be unbearable.

They suffer from homesickness as much as the people of other nationalities; but I have, whenever possible, allowed patients to go home for a brief visit, after which they have been contented to remain some weeks, even months, in the hospital.

Soon after opening the hospital, a man was brought in with a compound comminuted fracture of the humerus, and a compound fracture of the skull.

His life was despaired of, and my medical helper did not want us to admit him. He was really our first in-patient, admitted Feb. 14, 1910.

As he was in great pain, he was given a hypodermic of morphia sulphate gr. ¼ and atropia sulphate gr 1-150.

His wounds were caused by the explosion of a dynamite shell used by foreigners in blasting. He was 25 years old, and in good physical condition otherwise. His respiration was about 24, temperature 100:5 and his pulse 110. He was bleeding profusely, but the left radial artery was still beating, so that

I inferred the main brachial artery had not been severed. His left arm was very much swollen and in such a condition that I did not deem it wise to operate upon him in order to remove fragments of bone. I dressed the limb antiseptically, applied a temporary splint, and directed my attention to his head.

The right orbital plate and frontal bone were fractured, and the external wound communicating with it was dressed. Next day, I applied an internal angular wooden splint to the arm, so as to keep up extension and counter-extension. The case was a long-continued one. While the wounds of the head gave no great concern, the fractured humerus required constant attention. A counter incision was made for drainage, and the use of bichloride of mercury, peroxide of hydrogen and permanganate of potash solutions for irrigation. He had delayed union, so that direct fixation of fragments by Lane's bone ferrule was resorted to.

We made the bone ferrule of ox humerus. We boiled the bone, preparing it first by sawing, filing and polishing.

This case had an infection with suppuration. There was serious thought of amputation. When Dr. T. W. Ayers of Hwanghien, was asked to give his opinion, he gave a grave prognosis, and advised amputation. But, with my optimistic view, and the patient's family unwilling, we did not operate. His temperature at the time Dr. Ayers saw him, April 2nd, was 102.4°, and his strength had been undermined by confinement to his bed. The recumbent position was necessary up to this time on account of his head, which was dizzy when he sat up.

April 13, his wound was doing better and the bone was uniting. This was two months after the injury.

April 19.—His temperature rose and he had sweats. However, he soon improved enough to go home April 25, and has been improving ever since.

He lost flesh while in bed, but as soon as he could get up and about, began to gain again. When he went home he was out of doors much of his time, and when asked what he was doing, replied: "I am watching the chickens."

His color return and he was so hearthy and full in the cheeks that when I next saw him, I did not know him.

He had ample time in the hospital, so that after leaving for home, he came only occasionally for dressing his arm, and what was his surprise to find we had to take out a piece of bone which remained in for a month after he left the hospital. His arm had bony union of the fragments; but evidently the musuclo spiral nerve had been caught by the callus. I offered to operate and remove the offending callus, and extricate the ends of the nerve, but he did not consent. His arm was improving with exercise, and while still impaired in usefulness, has the bone solid throughout. I think the plate of bone helped to secure union, and thus, recovery.

Another compound comminuted fracture of even longer duration, was also treated. A Chinese man, aged 20, admitted March 5, was not finally dismissed until Sept. 17.

He had been run over by the wheels of a heavy cart, and both tibia and fibula were fractured in more than one place.

Direct fixation by two pieces of ox tibia and silver wire, March 14. Removed one-half of the ferrule of bone April 12th. He went home June 25. Returned July 20. July 22, anaesthetic: removal of Sener's ferrule and wire. Callus was detached by chisel and mallet. Able to walk.

He had a skin infection when I saw him again, Oct. 22, but was at work and able to make a living.

These two cases remained in the hospital without relatives or friends, and were nursed by our native helpers.

The bone plates were bored with holes by a bone drill, and silver wire was inserted as a means of securing the two plates.

Chang Tung Ke, aged 20, had elephantiasis, was under observation for a year, as an out patient. He first appeared lying in front of the church door, and begged for help. His left leg was twice the circumference of the right one, having thickened skin and the scales of rough and dry epidermis. The most prominent subjective symptom was itching, though some pain existed. He was given sulphur for relief of the itching and he himself applied it to the leg and foot.

This relieved the itching, and the leg was reduced in size considerably. The foot, which was thick and heavy, so that he lifted it with difficulty, was longer in yielding to treatment. He came for treatment when symptoms were urgent, and then when he was better left, pursuing his vocation, begging. He again appeared last summer on the streets as a beggar and was now unable to walk. He had infected foci on the thigh and leg. He was admitted as an in-patient at the hospital August 5, 1910.

We had him to comply with the usual routine, shave off his queue and take thorough bath, and discard his clothes for the hospital clothes. Then he was put in bed and kept in bed several weeks.

Treatment consisted internally of the use of ten minim doses of Donovan's solution three times daily. An ointment of equal parts of sulphur and mercurial ointments was applied to the leg and foot, with the firm support to both afforded by cotton lint and bandage.

His general appearance and health began to improve and the leg and foot to diminish in size. In a month's time his skin above the knee was quite pliable. Below the left knee the dry scales fell off and in two months he could walk very well.

He began to work his way after he could walk, and could help the gardener in drawing water and hoeing.

The disease had seemed to resist treatment more at the sole of the foot, but it became almost as tender as a babe's, when new skin took the place of scales and leathery bark.

During the course of his disease, he ate and slept well. His diet was simple and nourishing; and regular meals conduced much to his recovery. The most serious interruption in his progress was after three months' stay at the hospital. He then had a skin eruption above the waist. It was similar to herpes zoster, and was treated with collodion 1 dram, iodine scale 6 grains, carbolic acid 6 grains.

He was also given calomel and soda tablets. He recovered very well, and this skin eruption did not take on the form the other had, owing to prompt treatment.

He is still under treatment for elephantiasis, but has discontinued Donovan's solution, only having the ointment. He has been in charge of the gate at the hospital and is thoroughly reliable. While his leg is still thick, he has good use of it, and his foot is likewise useful.

The work of this hospital is carried on for the three-fold purpose of (1), alleviating suffering and curing disease; (2), teaching the Chinese sanitary living, with a model of cleanliness and asepsis; and (3), as an evangelistic agency in bringing men to Christ, their Savior.

It is a pleasure to feel that some progress has been made along all of these lines.

